# OGRO: The Overview of functionally characterized Genes in Rice online database

**DOI:** 10.1186/1939-8433-5-26

**Published:** 2012-09-24

**Authors:** Eiji Yamamoto, Jun-ichi Yonemaru, Toshio Yamamoto, Masahiro Yano

**Affiliations:** 1 National Institute of Agrobiological Sciences, 2-1-2 Kannondai, Tsukuba, Ibaraki, 305-8602 Japan; 2 National Institute of Agrobiological Sciences, 1-2 Ohwashi, Tsukuba, Ibaraki, 305-8634 Japan

**Keywords:** Rice (*Oryza sativa* L), Functionally characterized genes, QTL, Database

## Abstract

**Background:**

The high-quality sequence information and rich bioinformatics tools available for rice have contributed to remarkable advances in functional genomics. To facilitate the application of gene function information to the study of natural variation in rice, we comprehensively searched for articles related to rice functional genomics and extracted information on functionally characterized genes.

**Results:**

As of 31 March 2012, 702 functionally characterized genes were annotated. This number represents about 1.6% of the predicted loci in the Rice Annotation Project Database. The compiled gene information is organized to facilitate direct comparisons with quantitative trait locus (QTL) information in the Q-TARO database. Comparison of genomic locations between functionally characterized genes and the QTLs revealed that QTL clusters were often co-localized with high-density gene regions, and that the genes associated with the QTLs in these clusters were different genes, suggesting that these QTL clusters are likely to be explained by tightly linked but distinct genes. Information on the functionally characterized genes compiled during this study is now available in the O verview of Functionally Characterized G enes in R ice O nline database (OGRO) on the Q-TARO website (http://qtaro.abr.affrc.go.jp/ogro). The database has two interfaces: a table containing gene information, and a genome viewer that allows users to compare the locations of QTLs and functionally characterized genes.

**Conclusions:**

OGRO on Q-TARO will facilitate a candidate-gene approach to identifying the genes responsible for QTLs. Because the QTL descriptions in Q-TARO contain information on agronomic traits, such comparisons will also facilitate the annotation of functionally characterized genes in terms of their effects on traits important for rice breeding. The increasing amount of information on rice gene function being generated from mutant panels and other types of studies will make the OGRO database even more valuable in the future.

**Electronic supplementary material:**

The online version of this article (doi:10.1186/1939-8433-5-26) contains supplementary material, which is available to authorized users.

## Background

Rice is a model plant species for which many genetic and genomic resources have been developed. These resources include high-quality genome sequence information (Goff et al. 2002Yu et al. 2002International Rice Genome Sequencing Project 2005), high-efficiency transformation systems (Hiei and Komari 2008), bioinformatics tools and databases (reviewed by Nagamura and Antonio 2010), mutant panels (Chern et al. 2007; Miyao et al. 2007), and publicly available populations for genetic analysis such as b ackcross i nbred l ines (BILs) and c hromosome s egment s ubstitution l ines (CSSLs) (Fukuoka et al. 2010). These resources have contributed to remarkable advances in rice functional genomics during the last two decades, and many genes have been functionally characterized (Jiang et al. 2011). Because rice is an important food crop as well as a model plant, information derived from functional genomics research needs to be applied to rice breeding.

So far, most of the genomics research that has been applied to rice breeding has been related to q uantitative t rait l ocus (QTL) analysis, because, in many cases, agronomically useful alleles represent naturally occurring allelic variations that were identified as QTLs in cultivars, landraces, or wild species (Yamamoto et al. 2009; Xing and Zhang 2010; Miura et al. 2011). Information on rice QTLs from published articles has been compiled and is publicly available in the Gramene-QTL database (Ni et al. 2009); http://www.gramene.org/qtl/) and the QT L A nnotation R ice O nline database (Q-TARO; Yonemaru et al. 2010; http://qtaro.abr.affrc.go.jp/). Several of the genes responsible for QTLs have been cloned, but most have not yet been identified. Mapped QTL regions are often long enough to contain many genes, and introgression of such QTL regions may result in linkage drag, which results from the introgression of one or more unfavorable genes that are closely linked to the genes responsible for the target QTL. In cases where a QTL has been fine-mapped or the causal gene(s) have been identified, the problem of linkage drag can be overcome by means of marker-assisted selection of recombinants between the target gene or QTL and nearby unfavorable genes (Fukuoka et al. 2009>).

With the exception of genes that have been identified as those responsible for QTLs, most of the functionally characterized genes in rice have not been analyzed for allelic variation and functional differences in natural populations. However, such information is useful for QTL cloning using the candidate gene approach and for candidate gene association studies (Ehrenreich et al. 2009; Emanuelli et al. 2010). For these approaches, it is necessary to make the list of candidate genes involved in the trait of interest readily available for individual experimental design. It is also important that the genomic locations of functionally characterized genes can be readily compared with the location of QTLs involved in the same trait. Rice databases such as Gramene (Youens-Clark et al. 2011) and Oryzabase (Kurata and Yamazaki 2006) include information on gene function from published research. However, it is necessary to rearrange the data provided by these databases for carrying out the abovementioned approaches. We also found that several functionally characterized genes are not included in those databases, probably because information on such genes was published in agronomy and breeding journals rather than in genetics, genomics, or molecular biology journals.

In this study, our goal was to facilitate the application of gene function information to the study of natural variation in rice. To accomplish this, we comprehensively searched for articles related to rice functional genomics and established a list of functionally characterized genes. Information on each gene was summarized to facilitate direct comparison with QTL information from Q-TARO (Yonemaru et al. 2010). We also compared the genomic locations of functionally characterized genes and QTLs. The information on functionally characterized genes obtained in this study was compiled in a new database, the O verview of Functionally Characterized G enes in R ice O nline database (OGRO), which is located on the Q-TARO website (Yonemaru et al. 2010; http://qtaro.abr.affrc.go.jp/ogro).

## Results and discussion

### Extraction of information on functionally characterized genes in rice

**Figure 1:**
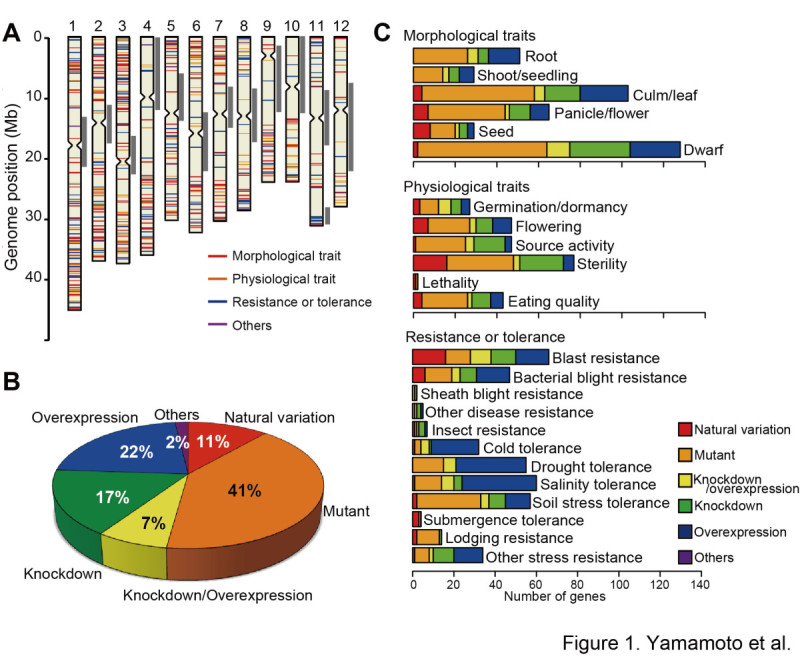
**Overview of the functionally characterized genes in rice.** (**A**) Genomic distribution of the 702 functionally characterized genes compiled during this study. The position of each gene is indicated by a horizontal bar; the color indicates the major category for that gene. Gray vertical bars to the right of each chromosome indicate heterochromatic regions (Cheng et al. 2001; Li et al. 2008). (**B**) The proportions of genes isolated by each method. (**C**) Numbers of functionally characterized genes in each trait category (total and by each of the methods listed in **B**).

To establish the list of functionally characterized genes in rice, we conducted a comprehensive search for articles related to rice functional genomics, and we extracted information on gene function by manually checking every article identified in the search. As of 31 March 2012, 702 functionally characterized genes were annotated based on the information from 707 articles. The categories of information extracted for each of the functionally characterized genes are listed in Table 1. The list of functionally characterized genes includes seven microRNAs (miRNAs) that have been associated with specific phenotypes (Xie et al. 2006; Zhu et al. 2009; Gao et al. 2010; Gao et al. 2011a). Figure 1A shows the genomic distribution of the 702 functionally characterized genes. Among these, four genes were absent from the reference genome sequence (*Oryza sativa* L. ssp. *japonica* cv. Nipponbare): *qSW5*/*GW5* for grain size (Shomura et al. 2008; Weng et al. 2008), *Sub1A* for submergence tolerance (Xu et al. 2006), and *SK1* and *SK2* for internode elongation in floating rice (Hattori et al. 2009). Genomic regions with few to no functionally characterized genes generally corresponded to heterochromatic regions (Figure 1A; Cheng et al. 2001; Li et al. 2008).

**Table 1:** **Information on functionally characterized genes extracted from each article**

Gene information item	Remarks
Gene	Unabbreviated gene name
Gene symbol	Abbreviated gene name
Major category	Corresponds to the criteria used in Q-TARO (Yonemaru et al. 2010; http://qtaro.abr.affrc.go.jp/)
Category of objective character	Corresponds to the criteria used in Q-TARO (Yonemaru et al. 2010; http://qtaro.abr.affrc.go.jp/)
Chr	Chromosome number
Genome start	Corresponds to IRGSP pseudomolecules build 4 (http://rgp.dna.affrc.go.jp/E/IRGSP/Build4/build4.html)
Genome end	Corresponds to IRGSP pseudomolecules build 4 (http://rgp.dna.affrc.go.jp/E/IRGSP/Build4/build4.html)
Locus ID	RAP locus (Rice Annotation Project 2008; http://rapdb.dna.affrc.go.jp/), MSU *Osa1* rice locus (Yuan et al. 2005; http://rice.plantbiology.msu.edu/), osa-miRNA ID (Griffiths-Jones et al. 2008; http://www.mirbase.org/), or GenBank (http://www.ncbi.nlm.nih.gov/genbank/) accession number
Method of isolation	The term "natural variation" was used for genes functionally characterized by using cultivars, landraces, or wild relatives. The term "knockdown/overexpression" indicates that the genes were characterized using both knockdown and overexpression transgenic plants.
Objective character	Phenotypes described in each of the articles
Reference	Identified by the Digital Object Identifier (doi)

There are 44 755 gene loci, excluding transposable elements (TEs) and ribosomal protein or tRNA loci, in RAP (Rice Annotation Project 2008; http://rapdb.dna.affrc.go.jp/), and 491 miRNA loci in release 18 miRbase (Griffiths-Jones et al. 2008; http://www.mirbase.org/). The functionally characterized genes compiled during this study represent only 1.6% of these loci. In Arabidopsis, a model dicot species, 5826 genes have been functionally characterized, accounting for more than 20% of the gene loci in this species (Lamesch et al. 2012). Considering both the number and the proportion of functionally characterized genes in Arabidopsis, it seems that the functional characterization of rice genes is far from complete.

For the gene information item "method of isolation" (Table 1), the genes identified by using cultivars, landraces, or wild relatives were described as "natural variation". Among the 702 functionally characterized genes, 11% (80 genes) had been identified through natural variation. Another 41% (286 genes) were identified by mutant analysis, and 48% (336) were identified by using transgenic plants (isolation method classified as "overexpression", "knockdown", "knockdown/overexpression", or “others”; Figure 1B). This breakdown indicates that both forward- and reverse-genetics approaches are valuable methods in rice functional genomics.

We annotated the functionally characterized genes based on the phenotypes described in each of the articles (Table 1). The phenotypes related to each gene were classified into "major category" and "category of objective character" (Table 1). These categories are identical to those used in Q-TARO (Yonemaru et al. 2010; http://qtaro.abr.affrc.go.jp/). Genes associated with multiple traits were counted within each relevant category.

The number of functionally characterized genes within each category is shown in Figure 1C. The variability in the number of functionally characterized genes among the different categories (Figure 1C) probably reflects the agronomic importance of each trait and the interests of individual researchers rather than the actual number of genes involved in each trait. In the major category "resistance or tolerance", transgenic approaches ("overexpression", "knockdown", and "knockdown/overexpression") were used for functional analysis more frequently than for genes in the major categories "morphological trait" and "physiological trait" (Figure 1C). This difference might be due to the difficulty in screening mutant and natural populations for traits related to resistance or tolerance. Within the major category "resistance or tolerance", most of the genes in the categories "cold", "drought", and "salinity" were characterized by overexpression analysis (Figure 1C). The overexpressing plants often showed pleiotropic effects such as growth retardation (Abbasi et al. 2004; Ye et al. 2009; Nakashima et al. 2007), suggesting that complex mechanisms control these abiotic stress tolerances in rice.

### Comparison of genomic locations between functionally characterized genes and QTLs

**Figure 2:**
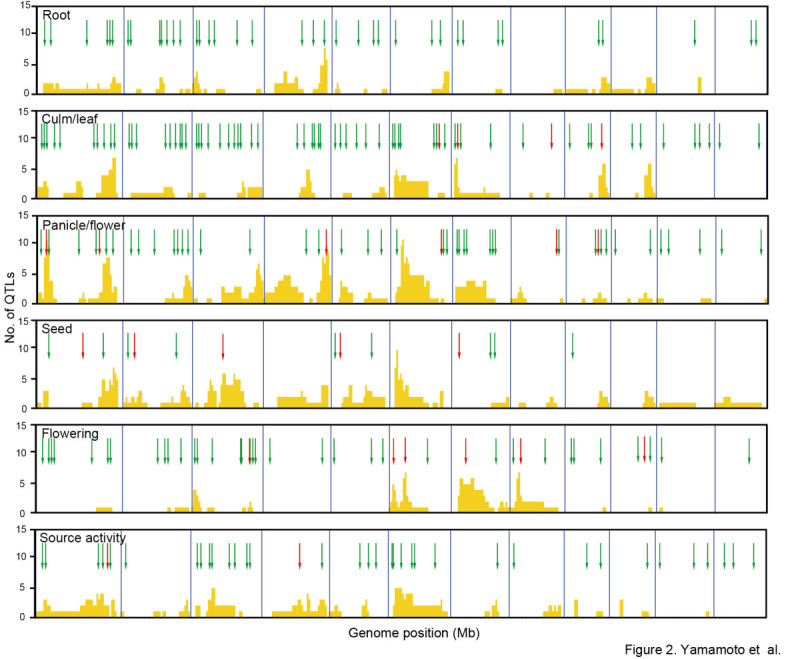
**Comparison of genomic locations between QTLs and functionally characterized genes within trait categories.** The categories chosen for comparison were those with sufficient numbers of both functionally characterized genes and QTLs. In each panel, the *x*-axis represents the complete 382-Mb genome of rice, with blue vertical lines marking the chromosome boundaries. Yellow bars indicate the number of QTLs within 1-Mb intervals. Red arrows indicate the genome position of genes identified by using natural variation. Green arrows indicate the genome positions of genes isolated by using mutant or transgenic plant analysis.

QTL analysis has been used extensively in rice to identify the chromosomal locations and phenotypic contributions of QTLs, and this information has been compiled in two databases, Gramene-QTL database (Ni et al. 2009; http://www.gramene.org/qtl/) and Q-TARO (Yonemaru et al. 2010; http://qtaro.abr.affrc.go.jp/). The gene function information gathered in the present study was arranged to enable direct comparison with the QTL information (trait classification and genomic location) in Q-TARO (Table 1). We performed a genome-wide comparison of the genomic locations of functionally characterized genes in several trait categories with those of the QTLs in Q-TARO (Figure 2). Because most of the genes functionally characterized by using natural variation were identified as genes responsible for QTLs, it was not unexpected to find that most of their genomic locations were included in QTL regions associated with those same traits (Figure 2). Moreover, the genomic locations of functionally characterized genes identified by mutant and transgenic plant analysis also showed good correspondence with QTL locations (Figure 2), indicating that these genes are good candidates for the genes responsible for the QTLs. The increasing amount of information on rice gene function will make the candidate gene approach for identifying genes responsible for QTLs even more powerful.

**Figure 3:**
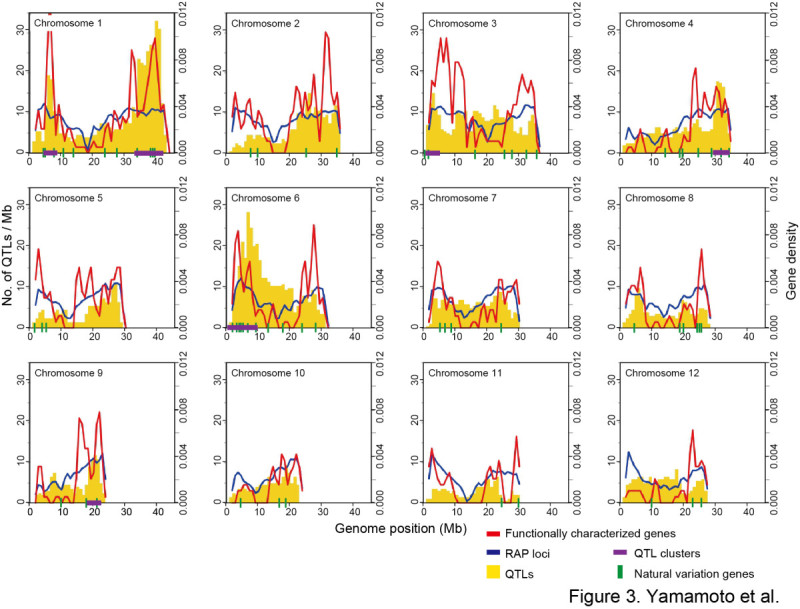
**Comparison of the density of functionally characterized genes and RAP loci and the number of QTLs.** The density of functionally characterized genes or RAP loci at each point in the genome is expressed as the proportion of the total number of genes (loci) contained within the surrounding 1-Mb block, calculated by using a window size of 2 Mb. The number of QTLs was counted within every 1-Mb block along the genome sequence. Red and blue lines indicate densities of functionally characterized genes and RAP loci, respectively. Yellow bars indicate the number of QTLs in each 1-Mb block. Green vertical bars on the *x*-axis indicate the position of genes categorized as "natural variation" in the method of isolation. Positions of QTL clusters are indicated by purple lines on the *x*-axis and are based on Yonemaru et al. (2010).

Many QTLs tend to be co-localized in specific genomic regions (QTL clusters) even though they control different traits (Yonemaru et al. 2010; Zhao et al. 2011). To survey whether functionally characterized genes were also arranged in such clusters, we calculated the distribution of functionally characterized genes and compared it with the genomic locations of the QTL clusters (Figure 3). In this comparison, we also included the gene density of RAP loci (Rice Annotation Project et al. 2008; http://rapdb.dna.affrc.go.jp/). There was good correspondence between the genomic locations of functionally characterized genes and RAP locus gene density (Figure 3). Furthermore, functionally characterized genes and QTLs also showed high co-localization (Figure 3), indicating that QTLs tended be located in regions of high gene density. Regarding the genetic basis of the QTL clusters, two main possibilities are generally considered: the pleiotropic effects of one or a few genes, or the effects of multiple genes that are tightly linked to one another. Several genes responsible for QTLs have been reported to have pleiotropic effects; for example, *SCM2* is involved in panicle architecture, culm length, and culm mechanical strength (Ookawa et al. 2010), and *IPA/WFP* is involved in panicle architecture, panicle number, and culm mechanical strength (Jiao et al. 2010; Miura et al. 2010). However, when we examined the genomic location of QTL clusters and genes identified by using natural variation, we found that the QTL clusters often contained multiple genes identified by using natural variation (Figure 3). For example, on the long arm of chromosome 1, which contains the largest QTL cluster region, there were four genes that had been identified by using natural variation: *Pi37* for blast resistance (Lin et al. 2007), *qSH1* for seed shattering (Konishi et al. 2006), *qNPQ1-2* for photosynthetic capacity (Kasajima et al. 2011), and *sd1* for culm length (Sasaki et al. 2002). On the short arm of chromosome 6, the location of the second-largest QTL cluster region, there were eight genes that had been identified by using natural variation: *wx* (Wang et al. 1995) and *alk* (Gao et al. 2011b) for eating quality, *Hd3a* (Kojima et al. 2002) and *Hd1* (Yano et al. 2000) for heading date, *DPL2* (Mizuta et al. 2010) and *S5* (Chen et al. 2008) for sterility, and *Pi2/Pi9* (Zhou et al. 2006) and *Pi25/Pid3* (Qu et al. 2005) for blast resistance. Although the genes responsible for most QTLs are still unidentified, considering these examples along with the data showing co-localization of QTL clusters and high-density gene regions (Figure 3) suggests that many QTL clusters are caused by groups of distinct but tightly linked genes controlling different traits. Understanding the cause of these QTL clusters is important for designing breeding strategies. If QTL clusters contain tightly linked but distinct genes, as in these examples, the problem of linkage drag can be overcome by repeated crossing and careful marker-assisted selection to break the linkage between desirable and undesirable genes and to select the ideal combination of alleles.

### Public database of functionally characterized genes in rice

**Figure 4:**
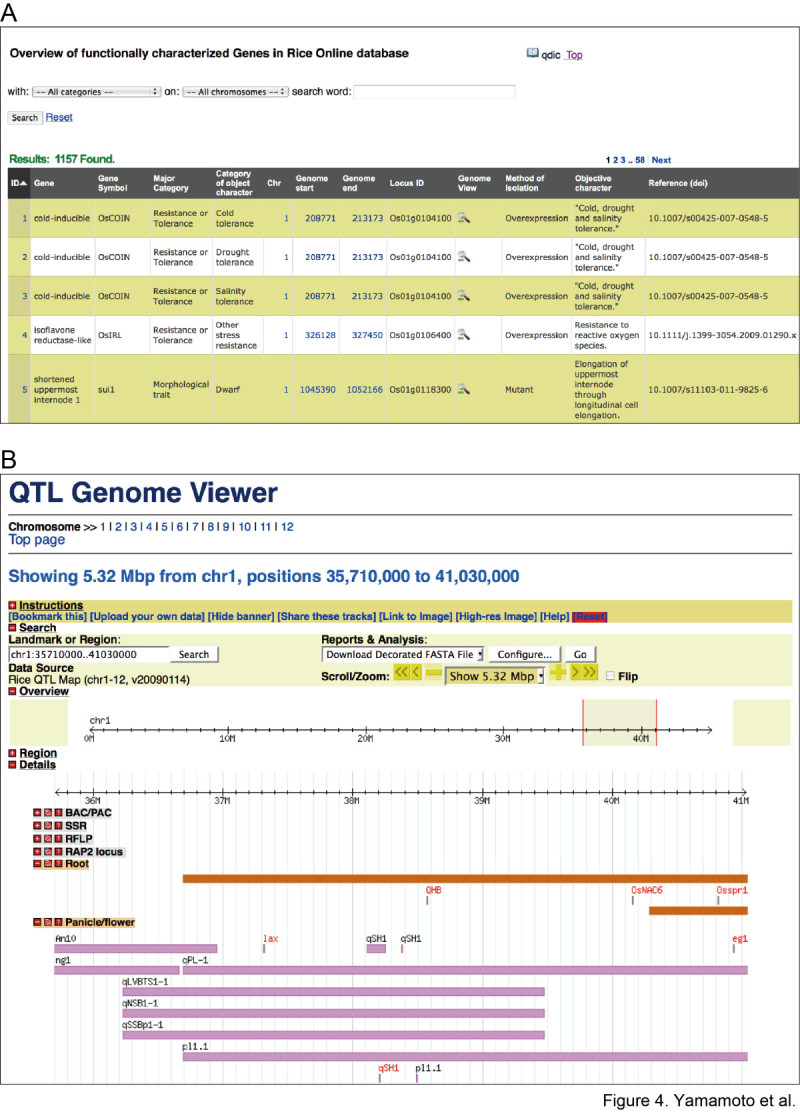
**Screen shots of the** **O** **verview of Functionally Characterized** **G** **enes in** **R** **ice** **O** **nline database (OGRO) (**http://qtaro.abr.affrc.go.jp/ogro**).** (**A**) Gene information table. All displayed information can be exported as comma-separated values (CSV format). (**B**) OGRO genome viewer. This viewer can be used to compare the locations of QTLs with those of functionally characterized genes.

OGRO is available on the Q-TARO website (Figure 4; Yonemaru et al. 2010; http://qtaro.abr.affrc.go.jp/ogro). Figure 4A shows a screen shot of an information table from the database. The desired information is displayed by selecting trait categories and chromosome locations from the drop-down boxes, or by specifying search text (Figure 4A, top). To view the genomic location of a target gene or to compare the genomic locations of genes and QTLs, the user can either specify the genomic location in the genome viewer or zoom in graphically (Figure 4B). By default, the loci are grouped by trait category, allowing the locations of genes and QTLs for each trait to be easily compared. Dragging and dropping of the horizontal information bars at the top of the window facilitates the comparison of genes and QTLs in different trait categories.

Although recent advances in next-generation sequencing technologies have enabled re-sequencing of a large number of rice genomes (Xu et al. 2011) as well as high-throughput genotyping and large-scale genetic variation surveys (McNally et al. 2009; Ebana et al. 2010; McCouch et al. 2010; Nagasaki et al. 2010; Yamamoto et al. 2010), analysis of gene function is still indispensable both for understanding fundamental phenomena and for genomics-based breeding. Increasing numbers of mutant panels have been developed in rice, and their comprehensive analysis is ongoing (Chern et al. 2007). These experiments will provide additional information on gene function, which will be added to the database as it becomes available.

## Conclusion

In this study, we comprehensively searched for articles related to rice functional genomics and extracted information on 702 functionally characterized genes (Figure 1). The information on each gene was organized to enable direct comparison with the QTL information in Q-TARO (Yonemaru et al. 2010; http://qtaro.abr.affrc.go.jp/), which will facilitate a candidate-gene approach to identifying the genes responsible for QTLs (Figure 2). Because the QTL descriptions in Q-TARO contain information on agronomic traits, such comparisons will also facilitate the annotation of functionally characterized genes in terms of their effects on traits important for rice breeding. We found that the genes responsible for QTLs in QTL clusters were identified as different genes (Figure 3). Considering this evidence along with the data showing co-localization of QTL clusters and high-density gene regions (Figure 3), our results suggest that many QTL clusters are caused by distinct but tightly linked genes. Information on the functionally characterized genes compiled in this study is now available in OGRO on the Q-TARO Web site (Figure 4; http://qtaro.abr.affrc.go.jp/ogro). The increasing amount of information on rice gene function being generated from mutant panels and other types of studies will make the OGRO database even more valuable in the future.

## Methods

### Extraction of gene information from published articles

Functional genomics studies have been done using many different approaches, and the degree of functional characterization differs substantially among genes. To avoid ambiguity, we established two main criteria for functionally characterized genes in rice. The first was verification of function: gene function had to be demonstrated in rice through direct evidence based on complementation tests, mutant analysis, or transgenic plant analysis. The second was verification of the phenotype: there had to be evidence that the function of the gene affected the phenotype of the rice plant. Functional analysis using other organisms such as yeast and Arabidopsis was not counted as meeting this criterion because such experiments do not necessarily indicate that the gene has a biological role in rice.

Articles related to rice functional genomics were identified by searching the Web of Science database (http://apps.webofknowledge.com/) with the search terms "rice" and "Oryza sativa". Because rice studies span a broad range of research fields, the following categories were surveyed: Agriculture Multidisciplinary, Agronomy, Biotechnology & Applied Microbiology, Cell Biology, Genetics & Heredity, Multidisciplinary Sciences, and Plant Sciences. To make this search comprehensive, the time span was set to "All" (i.e., all publications since 1899). As of 31 March 2012, we identified a total of 14 102 articles using these search conditions. All of the articles were then manually checked, and articles containing information on gene function that met our criteria for functionally characterized genes were selected. The result was a total of 707 articles. For each gene meeting the criteria for a functionally characterized gene, we extracted information including the gene locus ID, genome position, method of isolation, related traits, and reference information (doi) (Table 1). Whenever possible, the RAP ID number (Rice Annotation Project 2008; http://rapdb.dna.affrc.go.jp/) was used as the gene locus ID number. If there was no corresponding ID in RAP, the Michigan State University (MSU) locus number (Yuan et al. 2005; http://rice.plantbiology.msu.edu/) or GenBank (http://www.ncbi.nlm.nih.gov/genbank/) accession number was used. Information on genome position (start and end) was based on International Rice Genome Sequencing Project (IRGSP) Pseudomolecules build 4.0 (http://rgp.dna.affrc.go.jp/E/IRGSP/Build4/build4.html). The genome positions of genes not found in the reference genome (*Oryza sativa* L. ssp. *japonica* cv. Nipponbare) were indicated by using either a position adjacent to the deleted sequence or the positions of the flanking markers used for positional cloning. Under the method of isolation, "knockdown/overexpression" indicates that the genes were characterized by using both knockdown and overexpression transgenic plants.

### Comparison of genomic locations and densities between functionally characterized genes and QTLs

We compared the relative genome positions and distributions of functionally characterized genes and QTLs within each of the trait categories. The genome position of each functionally characterized gene was represented by the midpoint between the genome start and genome end positions (Table 1). The QTL information was extracted from Q-TARO (Yonemaru et al. 2010; http://qtaro.abr.affrc.go.jp/).

We also performed comparisons across all of the trait categories between the density of functionally characterized genes, the density of RAP loci and the number of QTLs. The density of functionally characterized genes or RAP loci at each point in the genome was expressed as the proportion of the total number of genes (loci) contained within the surrounding 1-Mb block, calculated by using a window size of 2 Mb. The number of QTLs was counted within every 1-Mb block along the genome sequence.

### Database construction

All data on the functionally characterized genes annotated in this study were compiled in OGRO (http://qtaro.abr.affrc.go.jp/ogro). Like Q-TARO (Yonemaru et al. 2010; http://qtaro.abr.affrc.go.jp/), OGRO consists of two Web applications: a gene information table and a genome viewer. The Web applications were implemented as Perl scripts and CGI modules. The database was constructed using MySQL, a relational database management system. We used the GBrowse viewer (http://gmod.org/wiki/Main_Page), which was configured to access OGRO from within the Q-TARO genome viewer.

## Authors' information

EY, JY, TY: National Institute of Agrobiological Sciences, 2-1-2 Kannondai, Tsukuba, Ibaraki 305–8602, Japan. MY: National Institute of Agrobiological Sciences, 1–2 Ohwashi, Tsukuba, Ibaraki 305–8634, Japan.

## Authors’ original submitted files for images

Below are the links to the authors’ original submitted files for images. Authors’ original file for figure 1 Authors’ original file for figure 2 Authors’ original file for figure 3 Authors’ original file for figure 4
