# Supplementary figures and images for: OGRO: The Overview of functionally characterized Genes in Rice online database
Source: Rice (N Y). 2012 Sep 24;5:26. doi: 10.1186/1939-8433-5-26 (PMC5520837; doi:10.1186/1939-8433-5-26)

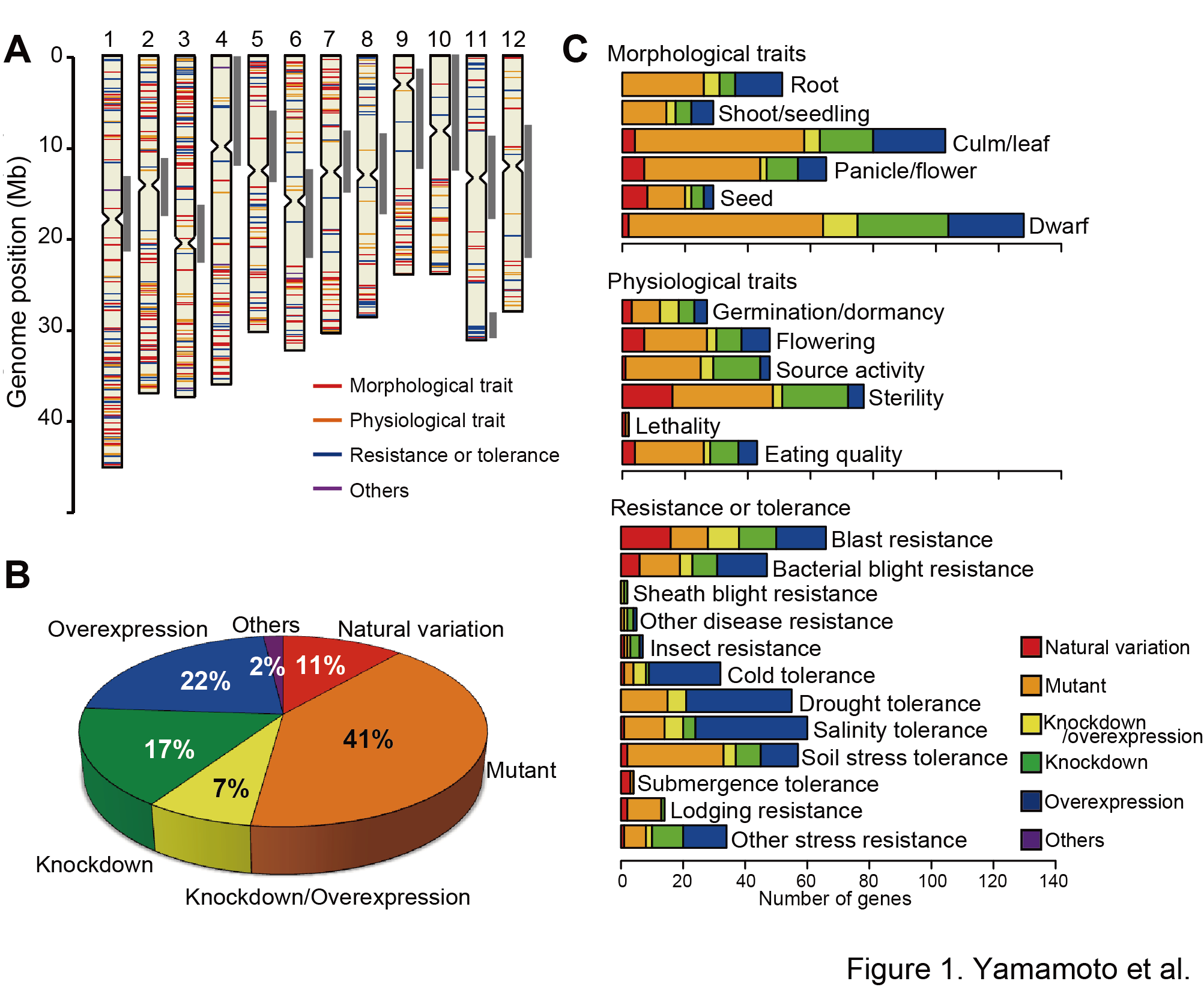

Supplement: Supplementary file 1 — Authors’ original file for figure 1 [file 12284_2012_20_MOESM1_ESM.tiff]

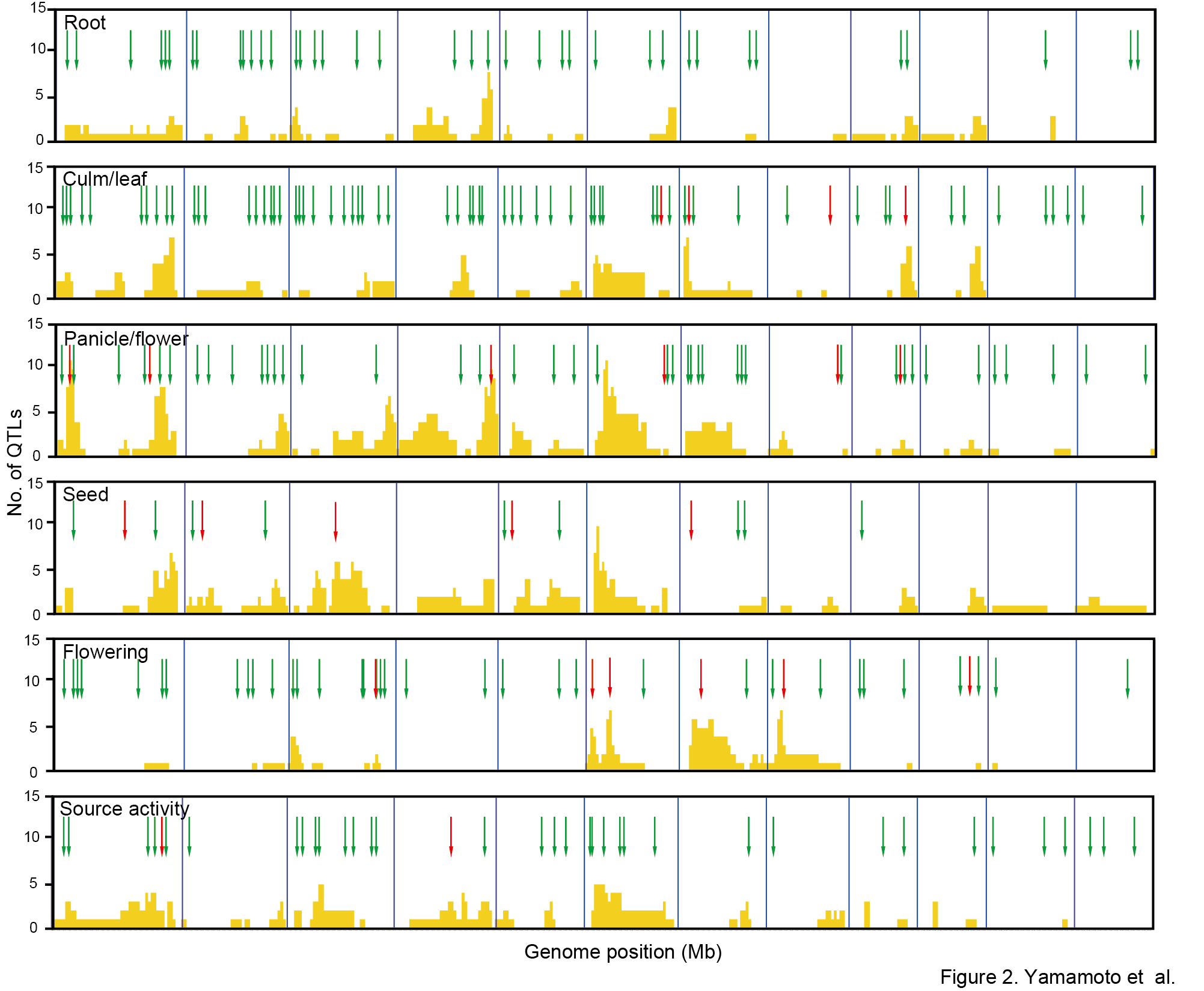

Supplement: Supplementary file 2 — Authors’ original file for figure 2 [file 12284_2012_20_MOESM2_ESM.tiff]

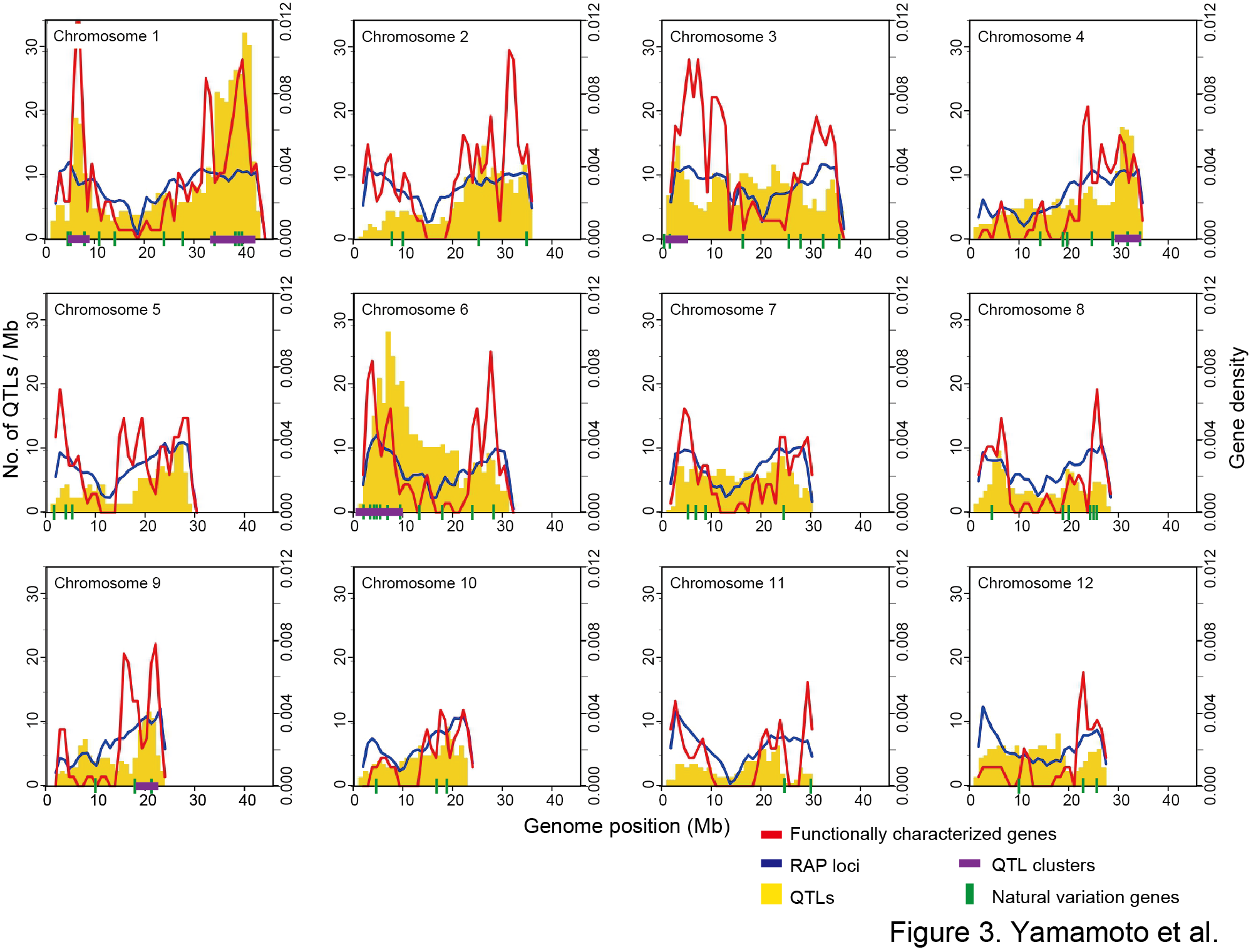

Supplement: Supplementary file 3 — Authors’ original file for figure 3 [file 12284_2012_20_MOESM3_ESM.tiff]

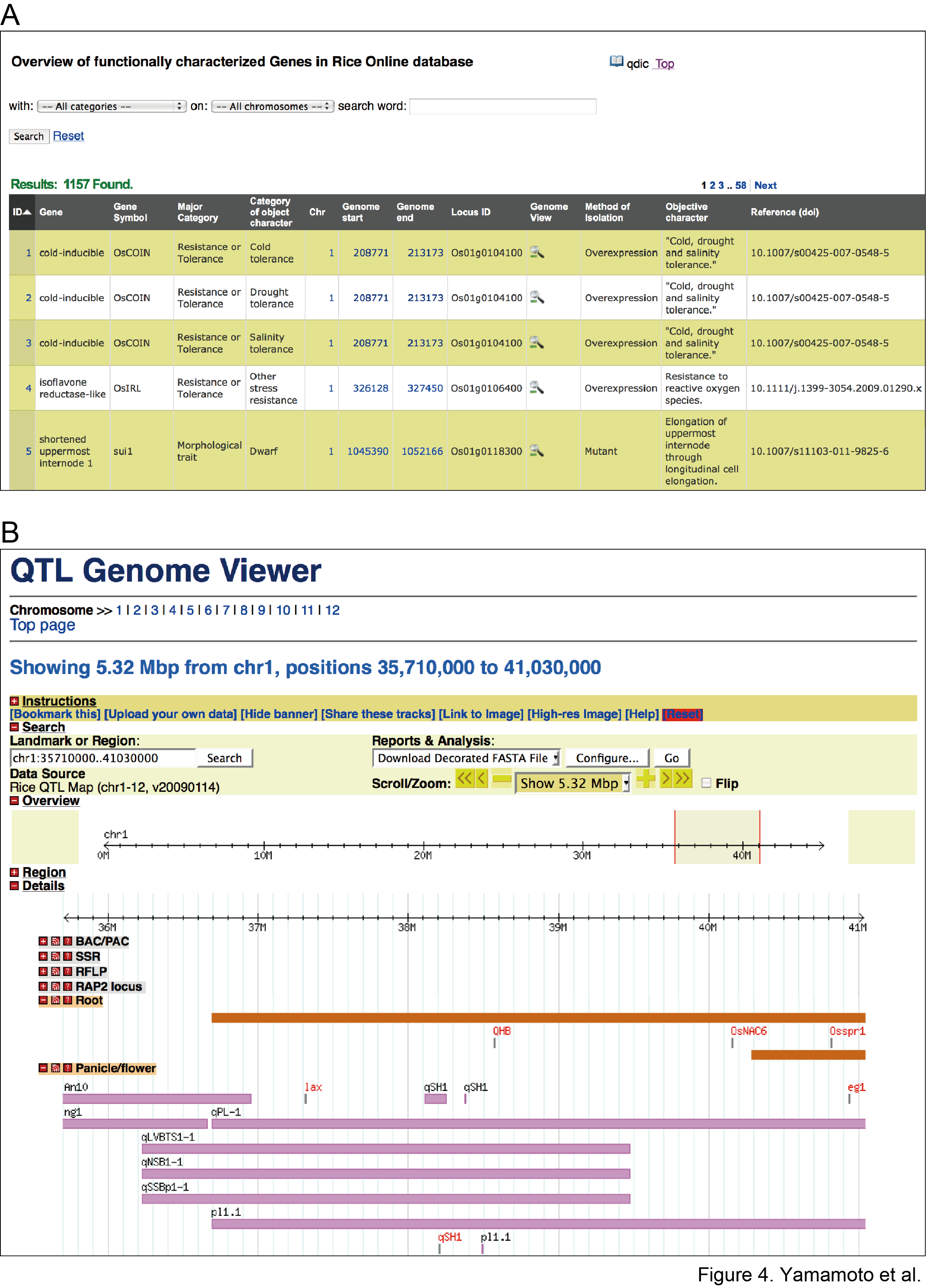

Supplement: Supplementary file 4 — Authors’ original file for figure 4 [file 12284_2012_20_MOESM4_ESM.tiff]
